# Interactions between Lateral Hypothalamic Orexin and Dorsal Raphe Circuitry in Energy Balance

**DOI:** 10.3390/brainsci14050464

**Published:** 2024-05-07

**Authors:** Vijayakumar Mavanji, Brianna L. Pomonis, Laurie Shekels, Catherine M. Kotz

**Affiliations:** 1 Research Service, Veterans Affairs Health Care System, Minneapolis, MN 55417, USA; mavan005@umn.edu (V.M.); pomon003@umn.edu (B.L.P.); sheke001@umn.edu (L.S.); 2 Department of Integrative Biology and Physiology, University of Minnesota, Minneapolis, MN 55455, USA; 3 Geriatric Research, Education and Clinical Center, Minneapolis VA Health Care System, Minneapolis, MN 55417, USA

**Keywords:** orexin, dorsal raphe, energy expenditure, obesity, age-induced, spontaneous physical activity

## Abstract

Orexin/hypocretin terminals innervate the dorsal raphe nucleus (DRN), which projects to motor control areas important for spontaneous physical activity (SPA) and energy expenditure (EE). Orexin receptors are expressed in the DRN, and obesity-resistant (OR) rats show higher expression of these receptors in the DRN and elevated SPA/EE. We hypothesized that orexin-A in the DRN enhances SPA/EE and that DRN-GABA modulates the effect of orexin-A on SPA/EE. We manipulated orexin tone in the DRN either through direct injection of orexin-A or through the chemogenetic activation of lateral-hypothalamic (LH) orexin neurons. In the orexin neuron activation experiment, fifteen minutes prior to the chemogenetic activation of orexin neurons, the mice received either the GABA-agonist muscimol or antagonist bicuculline injected into the DRN, and SPA/EE was monitored for 24 h. In a separate experiment, orexin-A was injected into the DRN to study the direct effect of DRN orexin on SPA/EE. We found that the activation of orexin neurons elevates SPA/EE, and manipulation of GABA in the DRN does not alter the SPA response to orexin neuron activation. Similarly, intra-DRN orexin-A enhanced SPA and EE in the mice. These results suggest that orexin-A in the DRN facilitates negative energy balance by increasing physical activity-induced EE, and that modulation of DRN orexin-A is a potential strategy to promote SPA and EE.

## 1. Introduction

Complex neural systems regulate energy homeostasis by exerting control over energy intake, physical activity and energy expenditure. These neural systems include hypothalamic sites such as the paraventricular nucleus, arcuate nucleus and the lateral hypothalamus (LH). Like the complex regulatory network for energy homeostasis, several brain sites and neurotransmitters influence SPA and EE. Although not comprehensive, the major neuropeptide systems that have been studied relative to SPA include cholecystokinin, brain-derived neurotrophic factor, corticotropin-releasing hormone, neuromedin U, neuropeptide Y, melanocortins, leptin, dynorphin, agouti-related protein, oxyntmodulin, orexins and ghrelin [1]. Though dopamine may be the final common signaling mechanism for the action of all of the above neuropeptides, other neurotransmitters such as acetylcholine, noradrenalin, serotonin and histamine are known to be involved in the regulation of motor function and EE [2]. Similarly, estrogen [3], adiponectin [4,5] and endocannabinoids are also suggested to be involved in the regulation of SPA and EE [2]. The neuropeptide best characterized in terms of its role in SPA and defense against obesity is LH orexin, also known as hypocretin [1]. LH orexin neurons are critical for the homeostatic and non-homeostatic control of energy balance and body weight regulation [1,6,7]. Orexin-A injected into various brain sites increases feeding [8], spontaneous physical activity (SPA) and thermogenesis [9,10,11,12,13,14] and orexin neuron ablation is associated with late-onset obesity and reduced locomotion, despite hypophagia [15,16,17,18]. Importantly, our earlier studies show that orexin injection stimulates SPA and associated non-exercise activity thermogenesis (NEAT) with a sustained promotion of total energy expenditure (TEE) [19], and orexin neuron activation increases NEAT, reduces obesity [20,21] and decreases body weight gain in animals [22,23]. Orexin can also promote feeding behavior, but this effect is short-lived, with compensatory feeding reduction in later time intervals, resulting in no overall effect on cumulative food intake [24]. In addition, our earlier studies have demonstrated no effect of orexin-A on energy intake when injected into some brain areas where it promoted SPA [8,13,25]. Together, these findings suggest that the SPA- and NEAT-enhancing effects of orexin-A are more closely aligned with improved energy homeostasis. The higher sensitivity of obesity-resistant (OR) rats to SPA promotion by orexin-A further supports the role of orexin in improved energy balance [11,26], but the underlying brain circuitry is less well understood. There is a neural connection from orexin neurons into the dorsal raphe nucleus (DRN), and thus, it is possible that orexin action in the DRN mediates the role of the DRN in energy homeostasis and the promotion of SPA and NEAT [1,7].

The DRN is a key site for the production and a major source of serotonin in the CNS, and serotonin is the primary neurotransmitter in the DRN [27]. The DRN is important for regulating behavior, appetite and thermogenesis [7]. Dysfunction of central serotonin (in the DRN and other areas) increases energy intake and body weight in humans and animal models [28]. On the contrary, elevating brain serotonin function is suggested as a potential therapy for metabolic disorders [29]. The DRN extensively projects into other cortical and subcortical areas involved in energy balance [7]. Orexin neurons send excitatory projections to the DRN, and both orexin receptors are expressed within the DRN [7]. Our earlier study demonstrated greater expression of orexin-1 receptor (OX1R) and orexin-2 receptor (OX2R) mRNA in the DRN of obesity-resistant (OR) rats [30]. Orexin administration into the DRN results in serotonin release in this site [31], thus identifying the DRN as a structure influencing the orexin-A-induced enhancement of SPA [30]. The DRN receives excitatory orexin inputs, and orexin modulates the serotonergic neurons of the DRN [30,32,33]. Studies have shown that non-resting thermogenesis, behavioral (SPA) sensitivity to orexin-A and OXR expression patterns (differential expression patterns of both OX1R and OX2R) in various brain areas including the DRN differentiate OR rats from control rats, thus identifying the DRN as a potential structure influencing the orexin-A-induced enhancement of SPA and EE associated with SPA, which is a subset of TEE [25,30]. Moreover, the administration of orexin-A into the rostral raphe pallidus enhances sympathetic outflow into brown adipose tissue (BAT) and BAT thermogenesis [34]. The DRN serotonin cells reciprocally innervate the LH orexinergic field and suppress the activity of orexin neurons [35,36], suggesting a feedback loop. Serotonin receptors are localized on the orexin neurons [35,37,38], and blunted serotonin transmission in the lateral hypothalamus and perifornical area is associated with weight gain and altered food intake and sleep/wake states, underscoring the complex role of orexin-DRN circuitry in energy balance (reviewed in [7]). Apart from directly exciting the DRN, serotonin–orexin interactions occur in other brain areas to modulate energy homeostasis [7]. Attenuation of serotonin neurotransmission through the genetic ablation of serotonin receptors results in obesity, eating disorders and sleep/wake disturbances, highlighting the role of orexin-DRN neurotransmission in energy homeostasis [39].

Subconsciously derived movement (spontaneous physical activity or SPA) and the EE resulting from it in kilocalories, known as “non-exercise activity thermogenesis” (NEAT), account for up to 30 percent of daily EE in humans [1], and it is a major determinant of individual susceptibility to diet-induced obesity [1]. Individuals and animals who increase their SPA and NEAT when overfed are shown to resist the development of obesity, suggesting that SPA and NEAT critically contribute to energy homeostasis and obesity resistance [7]. Spontaneous physical activity and programmed exercise are opposing extremes of physical activity in a continuum of intensity and structure. In humans, SPA includes fidgeting and time spent standing and ambulating to perform daily activities of living [1] and reflects an inherent propensity for PA rather than a goal-oriented activity [1]. Structured exercise is a goal-oriented activity and in rodents is best modeled by treadmill running [2]. The orexin system is central to the regulation of SPA, NEAT and energy balance [1]. Accordingly, we chose to measure spontaneous physical activity and associated energy expenditure in the current study.

The designer receptors exclusively activated by designer drugs (DREADD) method is a tool to achieve minimally invasive neuromodulation with cellular and anatomic selectivity. DREADD receptors are G-protein-coupled receptors (excitatory or inhibitory) modified to lose affinity to their biological ligand and undergo potent activation by a synthetic designer drug (or ligand). In this case, the designer drug was clozapine N-oxide (CNO), which is thought to be physiologically inert in rodents [20,40,41,42,43,44,45]. Orexin neuron-specific targeting was achieved in the current study via stereotactic injection of a virus containing the DREADD construct into the LH of mice engineered to express the DNA-recombinase Cre, driven by the orexin promoter. Thus, only orexin neurons express the Cre recombinase; therefore, the DREADD construct is expressed only in orexin neurons after viral construct injection. Accordingly, the systemically administered designer drug, CNO, selectively activates DREADD-expressing orexin neurons in LH [41].

Normal aging is associated with a reduction in SPA and EE and the development of obesity [46]. These metabolic changes observed in aging might be due to a reduction in orexin tone, as orexin system impairments/orexin neuron loss and a decrease in orexin peptide levels have been observed in both aged humans and animal models [46]. These data suggest that compromised orexin function is an important cause of age-related metabolic and behavioral disturbances. Accordingly, in this study, we determined whether the chemogenetic activation of orexin neurons ameliorates age-related decline in physical activity in middle-aged male mice. In an earlier study, we showed reduced OX neuron numbers in the LH of middle-aged (one-year-old) female mice, which was associated with a reduction in SPA and EE [46]. As central orexin or orexin neuron activation enhances SPA and EE, we aimed to determine whether orexin-A, administered directly into the DRN, could essentially “replace” the lost orexin tone in middle-aged animals and enhance SPA and EE. We targeted the DRN, based on its critical role in the regulation of motor function and EE. Given that orexin neurons project to and excite DRN neurons, and both orexin and DRN neurons are involved in the modulation of SPA, it is likely that serotonin neurons in the DRN are activated after chemogenetic stimulation of orexin neurons to enhance SPA. Accordingly, we aimed to test whether inhibiting the DRN (using GABAergic agents) blocks orexin neuron stimulation-induced SPA. The LH is a brain region that contains orexin-producing neurons. We injected the DREADD virus into the LH of orexin-Cre mice to activate these neurons and observe the effect of this activation on SPA and EE. As mentioned above, the DRN is important for regulating behavior, appetite and thermogenesis [7]. The orexin neurons project to and excite the DRN neurons, which also express both OX1R and OX2R receptors. It is unknown as to whether manipulation of GABA in the DRN alters orexin neuron activation-induced SPA, and if orexin-A injection into the DRN influences SPA and EE. Thus, to understand the role of DRN orexin in SPA and EE, we injected orexin into the DRN of mice. We hypothesized that (1) activation of orexin neurons enhances SPA in middle-aged mice and that DRN-GABA modulates the effect of orexin-A on SPA and (2) orexin injected into the DRN of middle-aged mice enhances SPA and energy expenditure. The current study shows that chemogenetic activation of LH orexin neuron stimulates SPA in middle-aged mice and that manipulation of GABA receptors in the DRN does not affect LH orexin neuron activation-induced SPA. Moreover, direct injection of orexin-A into the DRN of middle-aged mice enhanced SPA during the first hour after the injections, which resulted in enhanced EE. DRN orexin-induced SPA was positively correlated with EE, indicating that orexin neurons influence SPA and EE partly via serotonin neurons in the DRN.

## 2. Materials and Methods

### 2.1. Animals

This study was approved by the Minneapolis VA Health Care System Institutional Animal Care and Use Committee (protocol # VAM-19-00549; approval date 2 March 2020). We used adult (13 mo), male orexin–Cre (Orx/Cre) mice for the study. One-year-old mice have reduced OX neuron numbers [46] and SPA, and thus, we used this age group to determine if manipulation of the orexin system could improve SPA. The Orx/Cre mice were provided by Prof. Takeshi Sakurai (University of Tsukuba). They were bred in our mouse colony at the Minneapolis VA on a C57BL/6J background. The creation and phenotyping of Orx/Cre and WT heterozygous mice were performed as described earlier [41,47]. In the Orx/Cre mice, the orexin promoter controls the Cre recombinase so that only orexin neurons express the Cre recombinase. The mice were housed on a 12:12-h light–dark cycle with regular chow (3.1 kcal/g; 18.6% protein; 6.2% fat; 44% carbohydrates; Harlan Teklad 8604) and water available ad libitum

### 2.2. Virus Injection and Cannula Implantation

The animals were placed in a stereotactic apparatus (Kopf Instruments, Tujunga, CA, USA) following anesthetization with isoflurane (1–4%). Stereotaxic DREADD targeting was performed with a Cre-dependent adeno-associated virus (AAV) vector that expresses a double-floxed inverted open reading frame (DIO) near the DREADD transcript and a fluorescent tag (mCherry). The vectors (Addgene, Watertown, MA, USA) were stereotaxically administered into the LH (AP, −1.8/DV, −5.5/ML ±0.9 mm from bregma; bilateral, 333 nL/5 min/side) of the Orx/Cre mice. Excitatory DREADD-mediated neural activation was obtained via G_q_-coupled pAAV-hSyn-DIO-hM3D(G_q_)-mCherry (2.5 × 10^13^ GC/mL). During the surgical DREADD injection procedure, the mice were implanted with a unilateral 26-gauge stainless steel cannula (Plastics One, Roanoke, VA, USA) directed at the DRN (AP: −4.4/DV: −4.0/ML: ±0.27 mm from bregma). The injector extended 1.0 mm beyond the cannula tip. The animals were allowed a post-surgery recovery time of 3 wk. Following this, the mice were assigned randomly into appropriate groups to test them in the indirect calorimetry chambers. 

### 2.3. Drugs (CNO, OXA, Muscimol and Bicuculline)

CNO hydrochloride (CNO) is soluble in aqueous solutions and has no off-target effects in animals lacking DREADD receptors [48]. Accordingly, it is the first choice “designer drug”, with it being an inert and inactive clozapine metabolite [49], and it is used in the current study. The dosage used in the current study (1 mg/kg) is based on earlier studies from our and other laboratories [41,50]. We obtained water-soluble CNO dihydrochloride from Hello Bio (product # HB-6149, Princeton, NJ, USA). For SPA and EE measurements, 1 mg/kg of CNO (IP, dissolved in saline) or saline was administered using a small-gauge 3/10 CC insulin syringe 4 h post lights-ON (LightCycle). Acute 24 h tests were performed with a minimum of a 48 h washout period. 

In competitive binding assays, OX1R binds to orexin-A with high affinity, but it has considerably less affinity for orexin-B [51], suggesting that orexin-A is more selective for the OX1R. On the contrary, OxB is more selective to OX2R with one order (10-fold) of magnitude higher potency at OX2R compared to that at OX1R [52]. Sleep/wake states appear to be primarily regulated by OX2R and to a lesser extent by OX1R [53]. However, orexin-A (vs. orexin-B) has strong effects on physical activity, and a higher plasma orexin-A level was shown to be associated with physical activity in humans [54]. Animal studies also suggest a robust role for orexin-A (OXA) in the regulation of food intake and spontaneous physical activity (SPA) [55,56]. Orexin-A is more stable and more lipophilic than orexin-B [57]. Accordingly, we chose to use orexin-A in the current study. The orexin-A dose was based on our earlier studies showing behavioral effects in rodents following a 250 pmol dose of this compound [45,58,59]. In the current study, artificial cerebrospinal fluid was used to dissolve orexin-A (Sigma Aldrich, product # O6012), which was also used as the control (vehicle) for the DRN injections. The GABA-A agonist muscimol (product # M1523, Sigma Millipore, St. Louis, MO, USA) and the GABA-A antagonist bicuculline (product # 14343, Sigma Millipore, St. Louis, MO, USA) were dissolved in artificial cerebrospinal fluid. All drugs and stock solutions were stored at 4 °C prior to injections (for <48 h).

### 2.4. Metabolic and Behavioral Profiling (Indirect Calorimetry)

Spontaneous physical activity as measured by distance traveled and EE post injection were quantified using the Mouse Promethion indirect calorimetry caging system (Sable System™, Las Vegas, NV, USA). In this system, mice are housed in home-cage-like conditions with hanging food hoppers and water hoppers connected to inverted laboratory balances. Body weight throughout the test period was monitored using a third laboratory balance with a suspended empty plastic tube. Spontaneous physical activity was measured via infrared beam breaks in three axes: X + Y + Z. For indirect calorimetry, ambient air was passed through the cages (2  L/min) and gases were sampled from multiple points within the cage (250  mL/min). Raw data were collected using SableScreen v2.2 (SableSystem™) every second and extracted using Expedata v1.8.2 (SableSystem™). Energy expenditure was calculated using the respiratory quotient, Vmax O_2_/Vmax CO_2_, and converted to kCal. Macros available from the manufacturer (Sable Systems International, Las Vegas, NV, USA) were used to calculate total energy expenditure (TEE) using Weir equations [60]. The formula used by this macro is: EE [kcal/time] = VO_2_ [L/time] × (3.853 + 1.081RQ).

### 2.5. Experimental Design

All mice were injected with all compounds/combination treatments in separate experimental sessions. For the orexin neuron excitation (in mice injected with the excitatory DREADD construct) and DRN orexin injection studies, the test sessions started 3–4 h post lights-ON. As mice are not generally active during the light phase, we chose the post-lights-ON period (light phase) to increase their SPA via remote orexin neuron excitation or direct DRN orexin injection. In addition, in nocturnal rodents, orexin neurons were found to be highly active during their active phase (dark phase) [61]. Thus, injection during the light phase avoids a possible ceiling of orexin effects that could occur during the dark phase, due to higher endogenous orexin tone. The animals were first acclimated to the Promethion caging system (SableSystem™) for at least four days and then acclimated to the daily injection procedures for an additional 3 days (IP saline and DRN artificial cerebrospinal fluid (aCSF)). For testing, the mice were removed briefly, weighed and returned to the testing chamber for 24 h. Chow pellets and water were available ad libitum through hoppers in the chambers.

### 2.6. Orexin Neuron Excitation Study

Mice injected with the excitatory DREADD construct were given either IP vehicle or CNO (1 mg/kg) in a randomly assigned repeated measure design, 4 h after lights-ON, and then returned to the testing chambers immediately after the injections (to perform indirect calorimetry and SPA measurements) for ~24 h. The initial randomization of animals was based on their starting body weight distributions, the group averages of which were the same across treatments. Chow and water were available within the indirect calorimetry cages throughout the measurement period. The DREADD experiment was performed on ~13-mo-old Orx/Cre animals that were prepared with viral intracranial injections of pAAV2-hSyn-DIO-hM3D(G_q_)-mCherry. Mice injected with the excitatory DREADD construct were given either IP vehicle or CNO in a randomly assigned repeated measure design. Fifteen minutes prior to IP CNO or saline, the animals were infused with either the GABA agonist muscimol (32.5 pmol/0.2 μL), the GABA antagonist bicuculline (32.5 pmol/0.2 μL) or vehicle into the DRN in separate sessions, followed by measurement of SPA and EE. The muscimol dose was based on a study by Takahashi and colleagues [62], which showed that 6 pmol of muscimol enhanced alcohol-induced aggression in mice. At the end of the study, the animals underwent perfusion, and their brains were collected to confirm virus injection and cannula placement.

### 2.7. Direct DRN Orexin Injection Study

**Figure 1:**
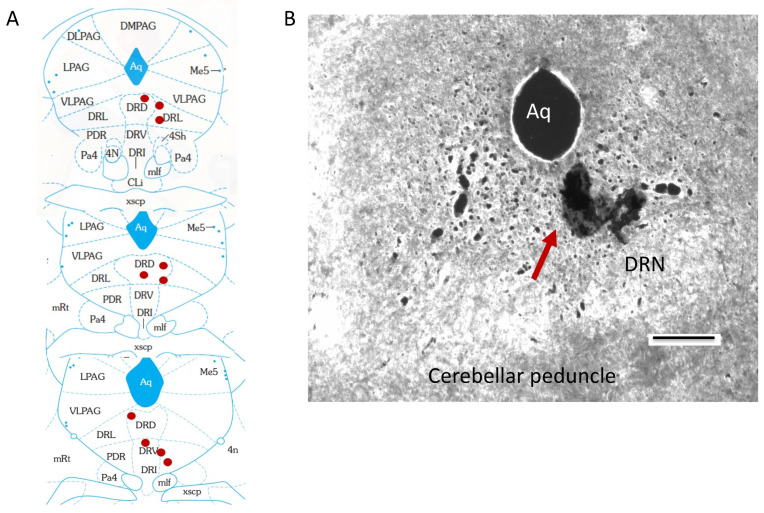
(**A**) Schematic representation of the injection location within the dorsal raphe nucleus (DRN). (**B**) The representative cryostat sections illustrate the presence of India Ink in the DRN following administration via the implanted cannula. DRD = dorsal DRN, DRV = ventral DRN, PDR = posterior DRN, DRL = lateral DRN and Aq = cerebral aqueduct. Scale bar = 750 μm.

Orexin tone in the DRN was manipulated via direct injections of orexin-A. Mice (n = 10, from the above study) with the cannula targeting the DRN were housed in the Promethion indirect calorimetry cages (SableSystem™) for continuous measurements of 24 h SPA and EE. Body mass and food consumption were measured every day. Either orexin-A (250 pmol/0.2 μL) or aCSF (consisting of 125 mM Nacl, 3 mM KCl, 2.5 mM CaCl_2_, 1.3 mM MgSO_4_, 1.25 mM NaH_2_PO_4_, 26 mM NaHCO_3_ and 13 mM D-Glucose) was administered into the DRN without any IP CNO injection to study the direct effect on SPA and EE of orexin injected into the DRN (for 24 h post injection). The DRN infusion of either 0.2  μL aCSF (Harvard Apparatus, Holliston, MA, USA) or orexin-A (250  pmol/0.2 μL aCSF) was performed using a syringe pump (KD Scientific Inc., Burlington, MA, USA) coupled to a Hamilton syringe (Series 7000, Sigma-Aldrich, St. Louis, MO, USA) and an injection assembly, consisting of flexible tubing and a 33-gauge injector (Plastics One, Roanoke, VA, USA), which extended 1.0 mm beyond the tip of the guide cannula [19]. The animals were gently restrained to place the injector into the DRN guide cannula, and the injection was performed over a duration of 30 s. The injector was left in place for an additional 10 s to ensure complete delivery and to minimize backward flow of the drug into the cannula tract. All injections occurred between 9 and 11 a.m. At least 48 h elapsed between treatments. An earlier study demonstrated that repeated injections performed in this manner do not cause tissue damage around the injection site even after 50 injections [63]. In addition, we did not observe any reduction in behavioral responses after repeated injections, indicating the preservation of tissue and cellular integrity following repeated injections. Cannula placement was confirmed through the use of light microscopy. Our histological analysis confirmed the correct targeting of the DRN in experimental mice used in the current study (Figure 1A,B). Based on the injection volume delivered and the diffusion coefficients, the injected OXA should remain confined to the DRN [64]. A randomly assigned Latin square unblinded design was used to evenly distribute the treatments. All mice were injected with all treatments (aCSF or OX) in separate experimental sessions.

### 2.8. Statistical Analysis

All data were analyzed using Prism 6.0 (GraphPad Software, San Diego, CA, USA). Statistical analyses of behavioral data and body mass were performed using Student’s *t*-test. For the DREADD study, the analyses of SPA and body mass were performed using two-way ANOVA followed by a Tukey’s post hoc test. For the hourly SPA data, two-way ANOVA followed by Tukey’s post hoc test was used to detect group differences. A simple linear regression was performed to determine the relationship between SPA and EE. All data are expressed as mean values ± S.E.M. An alpha level of 0.05 was used for all statistical tests. 

## 3. Results

### 3.1. Expression of DREADD-mCherry in Lateral LH Orexin Neurons

**Figure 2:**
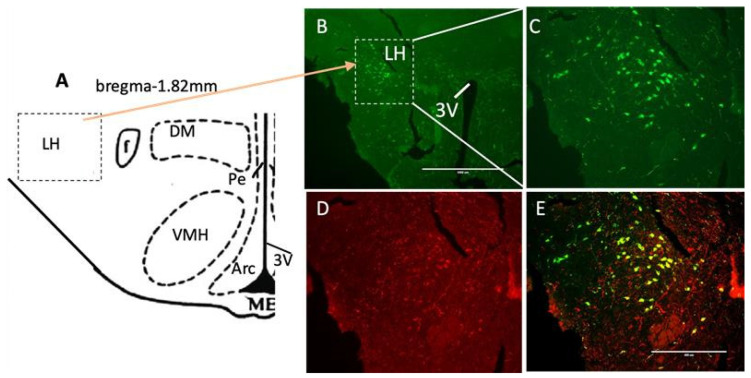
Chemogenetic targeting of orexin neurons. (**A**) Schematic representation of LH at −1.82 mm from bregma. (**B**) Photomicrographs of coronal sections containing immunofluorescent orexin neurons (green) in the LH (in high magnification; scale bar = 1000 μm). (**C**) Photomicrographs of coronal sections containing immunofluorescent orexin neurons (green) and hM3Dq-mCherry (red); (**D**) in the LH area. Orexin neurons expressing mCherry (orange) in Cre::hM3Dq mice (**E**). The lateral hypothalamus (LH), dorsal medial hypothalamus (DM), ventral medial hypothalamus (VMH) and third ventricle (3V). Scale bar in E = 400 μm.

A chemogenetic approach was used first to specifically activate LH orexin neurons using DREADDs, restricting expression to orexin cells in the lateral portions of the LH (Figure 2). Adult male orexin-Cre (Cre) mice received injections of a DREADD virus (hM3Dq) bilateral at one site per hemisphere. Immunohistochemistry (IHC) LH tissue analysis confirmed the selective expression of hM3Dq-mCherry in orexin neurons (Figure 2). The representative images show the orexin neuron field and clear colocalization of orexin (green, Figure 2B) and hM3Dq-mCherry (red; Figure 2C). The merged image in Figure 2D shows the expected cytoplasmic labeling of mCherry in orexin neurons (orange). 

### 3.2. Chemogenetic Activation of Orexin Neurons Enhances SPA in Mice

**Figure 3:**
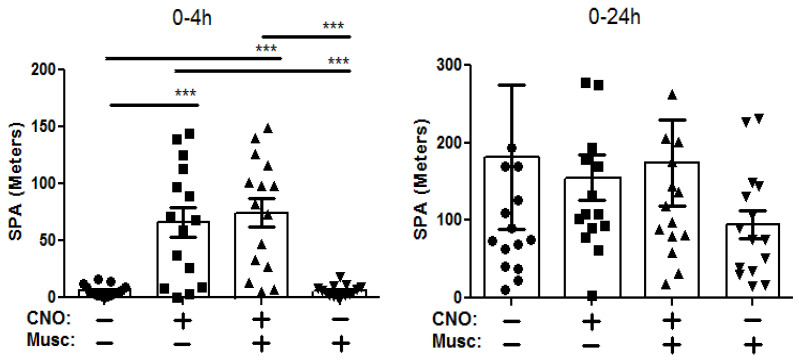
Acute pharmacosynthetic activation of orexin neurons increases SPA in male Cre::hM3Dq mice (13-mo, N = 16) at 4 h but not at 24 h after injection of CNO (1  mg/kg, IP). Data represent the means ± SE; *n* = 16/group. Groups: Mus (−)/CNO (−) = vehicle (aCSF, DRN) + vehicle (saline, IP); Mus (−)/CNO (+) = vehicle (aCSF, DRN) + CNO (IP); Musc (+)/CNO (+) = muscimol (DRN) + CNO (IP); Musc (+)/CNO (Mus) = muscimol (DRN) + vehicle (saline, IP). *** *p* < 0.001.

The mice prepared with the DREADD virus and injected with CNO (IP) exhibited significantly (*F*_3,56_ = 16.5, *p* > 0.001) greater SPA compared to the saline (IP)-injected mice up to 4 h post injection (Figure 3, Veh/CNO animals (65.98 ± 13.08, mean ± SEM) vs. Veh/Veh animals (6.98 ± 1.24), which were injected with vehicle into the DRN), without altering feeding behavior or body weight. These findings, along with our earlier published studies, demonstrate that orexin system activation is a potential avenue for stimulating SPA and associated EE. 

**Figure 4:**
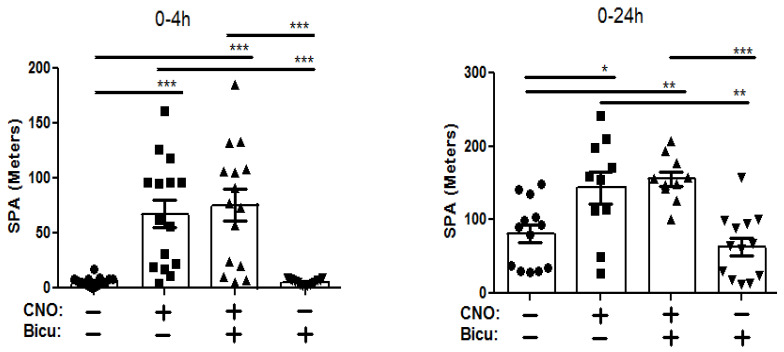
Acute pharmacosynthetic activation of orexin neurons increases SPA in male Cre::hM3Dq mice (13-mo, N = 16) at 4 h (left panel) and 24 h (right panel) after the injection of CNO (1  mg/kg, IP). Data represent the means ± SE. Groups: Bicu (−)/CNO (−) = vehicle (aCSF, DRN) + vehicle (saline, IP); Bicu (−)/CNO (−) = vehicle (aCSF, DRN) + CNO (IP); Bicu (+)/CNO (+) = bicuculline (DRN) + CNO (IP); Bicu (+)/CNO (−) = bicuculline (DRN) + vehicle (saline, IP). * *p* < 0.05, ** *p* < 0.01, *** *p* < 0.001.

The orexin neurons project to the DRN, and we next determined whether manipulating GABA neurotransmission in the DRN affects the SPA-enhancing effect of orexin neuron stimulation. Pretreatment with muscimol, (GABA agonist, Musc (+)/CNO (+) animals) directly into the DRN did not alter the CNO-induced (orexin neuron activation, Musc (−)/CNO (+) animals) enhancement of SPA at either 4 (*p* = 0.65, 74.44 ± 12.70 vs. 65.98 ± 13.08, Musc (+)/CNO (+) vs. Musc (−)/CNO (+) animals) or 24 h (*p* = 0.77, 174.44 ± 55.60 vs. 155.50 ± 29.07, Musc (+)/CNO (+) vs. Musc (−)/CNO (+) animals) post injection (Figure 3, left and right panel, respectively). Following this, we determined whether DRN administration of the GABA antagonist bicuculline influenced the increase in SPA observed with orexin neuron activation. Here also, the CNO-injected mice exhibited greater SPA (*F*_3,56_ = 16.2, *p* > 0.001) compared to the saline-injected mice up to 4 h post injection (Figure 4, left panel, Bicu (−)/CNO (+) animals (67.50 ± 12.66) vs. Bicu (−)/CNO (−) animals (5.96 ± 1.11)), an effect that was sustained over the 24 h measurement period (*F*_3,42_ = 16.5, *p* > 0.05; Figure 4, right panel, Bicu (−)/CNO (+) animals (143.80 ± 21.70) vs. Bicu (−)/CNO (−) animals (81.13 ± 12.42)). Like the effect of muscimol, pretreatment with the GABA antagonist bicuculline injected directly into the DRN did not alter the CNO-induced enhancement of SPA at either 4 (*p* = 0.67, 67.50±12.66 vs. 75.65 ± 14.80, Bicu (−)/CNO (+) vs. Bicu (+)/CNO (+) animals) or 24 h post injection (*p* = 0.63, 143.44 ± 21.69 vs. 155.7 ± 9.93, Bicu (−)/CNO (+) vs. Bicu (+)/CNO (+) animals). As behavioral changes occurred only during the initial 4 h, we chose to present this time point and the 24 data. Overall, these results indicate that GABA receptor manipulation in the DRN does not affect LH oractivation-inducedtion induced SPA. 

### 3.3. Injecting the DRN with Orexin-A Enhances SPA and EE in Mice

**Figure 5:**
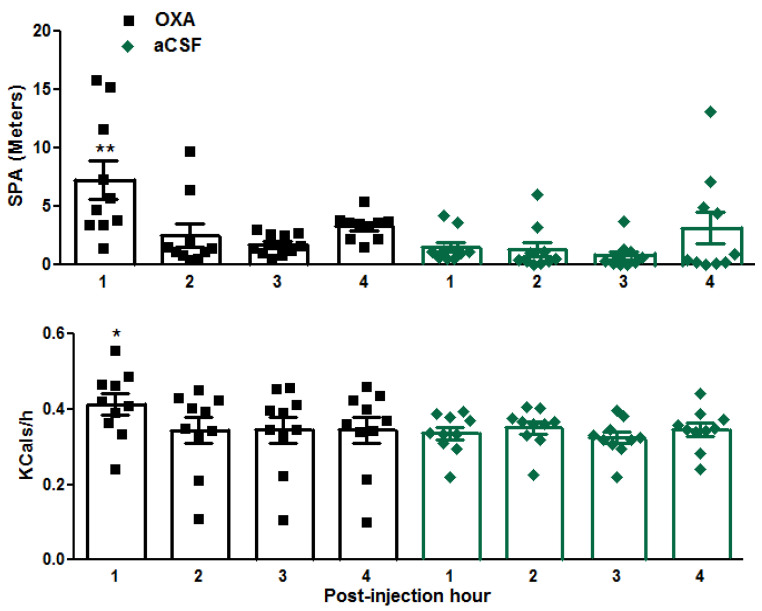
Acute DRN-injected orexin increases SPA and EE in male Cre::hM3Dq mice (13 mo, N = 10). The primary elevation in SPA and EE was observed during the first hour post injection. Data represent the means ± SE. Black lines and symbols represent data at hourly intervals following orexin-A injection (DRN), and green lines and symbols represent data at hourly intervals following aCSF injection (DRN). * *p* < 0.05, ** *p* < 0.01.

To determine the effect of direct administration of OXA into the DRN on SPA and EE, in a different study, either orexin-A (250 pmol/0.2 μL) or aCSF was administered into the DRN. The mice with orexin-A injected into their DRN showed enhanced SPA (meters traveled) during the first hour after the injections compared to that of the mice with aCSF-injected in their DRN (*p* = 0.003; 7.23 ± 1.63 vs. 1.51 ± 0.42, OXA vs. aCSF animals; Figure 5 top panel). Similarly, 24 h SPA was not significantly different between the orexin-A-injected vs. aCSF-injected mice (*p* = 0.113; 65.82 ± 8.57 vs. 47.16 ± 7.20, OXA vs. aCSF animals). Reflecting its effect on SPA, orexin-A injected into the DRN enhanced EE during the first hour after the injections compared to that after aCSF injection (*p* = 0.03; 0.41 ± 0.03 vs. 0.34 ± 0.16, OXA vs. aCSF animals; Figure 5, bottom panel). The 24 h EE was not significantly different between the orexin-A-injected vs. aCSF-injected mice (*p* = 0.11; 7.84 ± 0.02 vs. 7.90 ± 0.03, OXA vs. aCSF animals). Similarly, 24 h food intake and body weights were not significantly different between the orexin-A-injected vs. aCSF-injected mice). The plasma half-life of orexin is short (27.1 ± 9.5 min, [65]). Accordingly, it is possible that the transient effect of OXA injected into the DRN was due to rapid metabolization of the drug.

### 3.4. Enhanced SPA Is Positively Correlated with Increased EE

**Figure 6:**
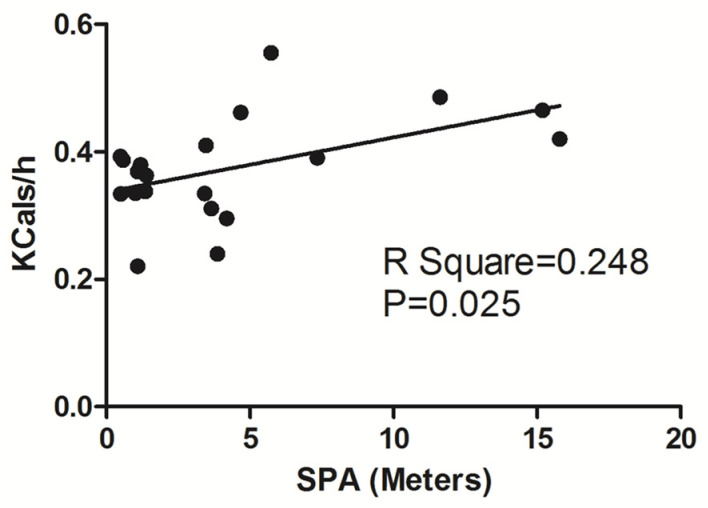
Regression analysis of spontaneous physical activity (SPA) and energy expenditure (EE) in the 1-h post-injection period, including animals from all treatment groups (n = 20).

We next determined whether there was a correlation between the increase in SPA and EE during the post-injection hours (1–4) using simple linear regression. Regression analysis comparing SPA and EE indicated that SPA was positively correlated with EE (*R*^2^ = 0.247, *p* = 0.025; Figure 6), indicating that greater time spent moving during the first hour following orexin-A administration resulted in enhanced EE. Interestingly, during the 2–4 post injection hours, this correlation was absent (*R*^2^ = 0.046, *p* = 0.362; *R*^2^ = 0.0001, *p* = 0.965 and *R*^2^ = 0.242, *p* = 0.512 for post-injection hours 2, 3 and 4 respectively). 

## 4. Discussion

The current study demonstrated that the chemogenetic activation of LH orexin neurons stimulates SPA, with it not being inhibited by the manipulation of GABA receptors in the DRN. Direct injection of orexin-A into the DRN enhanced SPA during the first hour after injection, which resulted in enhanced EE during the same time period. The DRN orexin-induced SPA was significantly and positively correlated with EE.

Orexin-A is a neuropeptide that integrates numerous physiological functions, promotes wakefulness, stabilizes sleep/wake states and enhances SPA and EE. Earlier studies have demonstrated enhanced SPA, EE and weight loss following the central administration of orexin or the chemogenetic activation of orexin neurons [20,41,46,66]. Earlier studies from our laboratory have demonstrated the enhancement of SPA following the chemogenetic activation of orexin neurons in young animals (~5-month-old mice) [20,41,46]. In one of the studies, middle-aged female mice moved less, and their SPA increased and reached the level of young animals after orexin neuron activation [46]. Orexin-A action on SPA and EE following its administration into the DRN has not been well studied thus far. The current results demonstrate that the activation of orexin neurons or localized administration injection of orexin-A into the DRN produces behavioral effects like those observed after the administration of orexin-A via injection into other central nervous system targets. Microinjection of orexin-A into the DRN enhanced SPA and energy expenditure without changing food intake or body weight. In addition, our results indicate that manipulating GABA-A receptors in the DRN does not affect orexin-A-stimulated SPA. Together, these results suggest that the DRN may be an important brain area through which orexin-A mediates SPA and energy balance homeostasis but not via GABA-A-responsive neurons.

The DRN expresses both OX1R and OX2R orexin receptor subtypes, and there are reciprocal connections between orexinergic LH orexin neurons and the DRN [7]. Orexins excite dorsal raphe serotonin neurons [67,68,69], an effect that is reversed by knocking out both orexin receptors [70,71,72]. A study from our laboratory also showed the presence of both OX1R and OX2R in the DR of rats [30]. Earlier findings [68] demonstrated a monosynaptic connection from the LH orexin field to the DRN. Earlier neuroanatomical, computational and functional data indicate that the DRN neurons are regulated by LH orexin, which in turn inhibits the LH orexin neuron cells [67]. Thus, LH orexin neurons can stimulate the DRN neurons to promote SPA and EE and can be inhibited by DRN serotonin neurons in a feedback manner. The increased SPA and EE observed in the current study agree with earlier findings that attribute a motor function to the DRN. The DRN neurons project onto forebrain areas involved in feeding and energy homeostasis. In addition, the DRN heavily projects into cortical and subcortical areas involved in movement preparation and execution such as the basal ganglia, motor cortex, cerebellum and spinal cord [7,73]. Thus, orexin may exert its modulatory role on physical activity via its projections into the DRN and other areas critical for motor function [74]. A series of studies support the role of the DRN in motor control. For example, greater serotonin release is observed in the dorsal but not medial raphe nucleus following orexin administration into these areas [31]. Changes in DRN serotonin levels affect motor behavior [75], and in movement disorder patients, the basal ganglia and cerebellum exhibit lower levels of serotonin [73]. Serotonergic drugs are used to treat motor diseases such as Parkinson’s disease [73], and the neural activity of DRN serotonin neurons correlates with muscle tone and the intensity of locomotor activity in cats [73]. Similarly, spinal cord motor neurons are excited by serotonin, which enhances locomotor activity [73,76]. Furthermore, injecting GABA into the DRN elicits depression-like behavior in mice [27], and DRN serotonin cells affect multiple behavioral parameters, such as sensory input, motor output and reward. In addition, DRN serotonin, along with other neuromodulators, elicits cortical desynchronization necessary for the awake state, which is critical for motor behavior [77]. Moreover, a deficient serotonin system results in impaired behavioral control [78,79,80,81]. On the other hand, orexin-A strongly excites DRN serotonin neurons [69,78], and 5HT2A and 5HT2C receptor antagonists attenuate orexin-induced behaviors (such as grooming) [82,83,84], demonstrating that the behavioral effects of orexin are partly mediated by serotonin. Our results further support a role for orexin–DRN interactions in enhancing motor behavior, as SPA and EE were elevated following the administration of orexin into the DRN. These findings, together with those studies showing higher SPA levels and elevated OXR1 and OXR2 mRNA in the DRN [30] and altered serotonin turnover in OP rats [85], further demonstrate an important role for the DRN orexin promotion of SPA and EE.

In contrast with the effect of the chemogenetic activation of orexin neurons or orexin-A administration into the DRN on SPA and energy expenditure, these manipulations failed to augment 24 h feeding behavior (so as not to disturb animal behavior following injection, we did not measure acute food intake in these experiments). This is in alignment with earlier observations that orexin-A exerts a brain site-specific influence on feeding [25]. In contrast with the enhanced food intake following orexin-A injection into the rostral portion of the LH [8], there was no effect of orexin-A on food intake following orexin-A injection into the locus coeruleus, substantia nigra, ventrolateral preoptic area or tuberomammillary nucleus [13,25,66]. The effects of orexin-A on food intake are distinct from other orexin functions such as sleep/wake states and SPA. These findings are in agreement with earlier studies demonstrating that orexin-A behavioral effects are brain site-specific [74], and expected, as orexin-A neurons integrate physiological responses to bodily disequilibrium caused by physical activity, hunger, metabolite accumulation or dietary changes [86,87], and orexin neuron firing is modified by alterations in pH, ATP, leptin, ghrelin, glucose and insulin [88,89,90].

The mechanisms by which orexin-A in the DRN modifies behavior are yet to be understood. Though DRN neurons receive GABAergic projections from other areas, a major source of GABA in the DRN is the local neurons characterized within the DRN. Some of these GABAergic neurons are interneurons and others are projection neurons [91]. Though orexin stimulates DRN serotonin neurons, it also increases impulse flow in local GABA interneurons. Accordingly, orexin is found to increase the basal firing of presumptive DRN GABA interneurons. Immunolabeling shows that orexin fibers project to both serotonin and GABA neurons in the DRN. It was suggested that orexin acts directly to excite DRN serotonin neurons with high potency and indirectly to activate local inhibitory GABA inputs to these serotonin cells. The greater potency of orexins in direct excitation compared with indirect inhibition suggests a negative feedback function for the latter at higher levels of orexin activity [92]. In addition, a recent study demonstrated the presence of a group of temperature-sensing GABAergic (Vgat-expressing) neurons in the dorsolateral portion of the DRN [93]. These GABAergic cells are stimulated by ambient heat and modulate thermogenesis by altering locomotor behavior and heat production via projections to brown fat and areas important for the central regulation of locomotion [93]. Optogenetic stimulation of these neurons decreased locomotion and thermogenesis, which aligns with the current study, in that stimulation of DRN by orexin enhanced SPA and EE. Orexin-A increases local glutamate and/or GABA release and can stimulate or inhibit target populations [94,95], providing a basis for the idea that orexin may inhibit these Vgat-expressing DRN neurons, to disinhibit their target areas and enhance SPA and EE. The DRN projects to other brain areas involved in locomotion, such as the motor cortex, basal ganglia, cerebellum and spinal cord. Thus, another possible mechanism for the modification of SPA and EE by DRN orexin-A is the activation of DRN neural efferent projections to these locomotor regulatory areas. 

One surprising observation in the current study is that direct manipulation of DRN GABA-A neurotransmission did not affect OXA-induced changes in EE or basal SPA. It is possible that this is because the DRN inhibits the LH [35,36,67,96], and the administration of a GABA agonist into the DRN leads to disinhibition of the LH, resulting in no decrease in orexin neuron stimulation-induced SPA. The GABA antagonist was expected to activate DRN neurons, which in turn should inhibit orexin neurons and thus reduce SPA. However, the GABA-A antagonist bicuculine failed to reduce the increase in SPA induced by orexin neuron stimulation. This may be because the chemogenetic activation of orexin neurons overrides inhibitory input from the DRN, and thus activation of DRN by the GABA antagonist did not affect SPA induced by orexin neuron stimulation. Moreover, as discussed in the previous paragraph, the DRN serotonin neurons are tightly controlled by intrinsic orexin and GABA mechanisms. Accordingly, the external GABA agonists or antagonists in the current study did not alter orexin neuron activation-induced SPA, which suggests the involvement of other brain regions in the orexin stimulation of SPA. 

In the current study, the chemogenetic activation of orexin neurons or direct orexin injection into the DRN enhanced SPA. This agrees with earlier studies showing that selective serotonin reuptake inhibitors (SSRIs) reduce immobility in a forced swim test and a tail suspension test [97,98]. Similarly, stimulation of the prefrontal cortex projecting-dorsal raphe nucleus promoted locomotion [97], and enhanced 5-HT biosynthesis did not initiate mood impairment or central fatigue [99]. On the contrary, several studies suggest a movement-inhibitory role for 5-HT neurotransmission. For example, elevated brain 5-HT is associated with reduced motivation for physical activity, depression and lower exercise output, which hastens central fatigue [100,101]. Similarly, an association between lower concentrations of 5-HT in several brain regions and higher exercise output and delayed fatigue has been demonstrated [100,102,103]. Furthermore, serotonin production was higher in a rodent model of exercise-induced fatigue [104], whereas 5-HT receptor antagonism in humans and animals delayed fatigue and improved endurance [105,106]. Surprisingly, a pharmacological study in humans failed to alter exercise capacity through changes in serotonergic neurotransmission [107]. The above-mentioned studies indicate a complex role of 5-HT in locomotion. Serotonin is important for the control of the intensity of locomotor activity [73] and the modulation of motor behavior based on the level of environmental stress [97], the intensity of exercise [108] and the exercise duration [109]. For example, serotonin suppresses movement in low- and moderate-threat environments but induces escape behavior/movement in high-threat environments [97]. Another study showed that a 6 h treadmill exercise induced central fatigue in rats compared to short-duration exercise [110]. It has been proposed that the prolonged release of serotonin during bouts of motor activity spills over from its release sites to the axon initial segment of the spinal motoneurons, where it activates inhibitory 5-HT1A receptors and inhibits the firing of motor neurons and muscle contraction, resulting in fatigue [105,106,109,110,111]. It is also suggested that in over-trained athletes, central fatigue, mental deficiency and behavioral alterations might be initiated by a central exhaustive exercise stress which elicits the impairment of complex neuromodulation in addition to the serotonin system [99]. Furthermore, the serotonin neurons are diverse in function (some 5HT neurons control respiration), and central fatigue under extreme exercise conditions might be an adaptive mechanism to prevent the respiratory system from over-exhaustion [112]. Overall, high serotonin-induced fatigue occurs only when the organism is subjected to a prolonged duration of exercise compared to short-duration exercise [110] or when subjected to maximal exercise capacity (compared with submaximal exercise) [108,111,113,114,115]. However, in the current study, orexin DRN enhanced SPA only during the first hour post injection, ruling out any effect of central fatigue in our study. In addition, we have recently demonstrated that the central injection of orexin enhances the excitability of the motor system in the brain [116], suggesting that DRN orexin-induced enhancement SPA may result from heightened brain motor preparedness. Overall, 5HT signaling has a complex role in the modulation of motor behavior and the current data suggest that the orexin–DRN pathway positively modulates SPA. 

There are a few limitations of this study. Based on our [41] and others’ previous findings, we do not think that the injection of CNO into non-DREADD-prepared mice would alter the behavioral outcomes, but we did not include a group of non-DREADD mice injected with CNO. Previous studies have shown that non-DREADD-expressing (control) mice do not show any behavioral or thermogenic effects after either IP CNO or saline (vehicle) injections, whereas DREADD-expressing mice injected with CNO showed changes in behavior [43,44,45] and thermogenesis [93], confirming that the observed effects in the current study are due to specific orexin neuron activation by CNO in DREADD expressing animals. We also did not measure the spread of the microinjected pharmacological agents, but Nicholson’s work showing that interaction between the ligand and receptor limits diffusion suggests that it is unlikely that appreciable or effective amounts extend beyond the DRN [64]. Another limitation is the lack of data showing cFos expression following pharmacological manipulation in mice. However, the mCherry/DREADD transfection in the orexin neurons was successful in the current study (Figure 2), which is consistent with our earlier publications that consistently confirmed selective colocalization of hM3Dq-mCherry in orexin neurons [20,41,43,44,45,46,117]. In addition, our earlier study reported mCherry expression in 69.9% of orexin neurons in orexin-Cre mice, which was significantly greater than the 0% of cells infected in the wild-type mice. In that study, CNO induced significant expression of cFos in the LH and achieved activation in about 73.7% of orexin-labeled cells [41], indicating the high specificity and efficacy of Cre expression in the orexin–Cre mice. Accordingly, we would expect neural activation in the LH and DRN following the chemogenetic activation of orexin neurons or the DRN injection of orexin [41,43,44,45,46,118]. An additional limitation is the lack of dose–response data on the GABAergic agents, but the dose used in the current study was based on an earlier study [62]. The lack of a dose–response study related to orexin-A in the DRN and SPA/EE might also be considered a limitation, but the orexin-A dose used was based on our earlier dose–response studies showing behavioral effects in rodents following a 250 pmol dose of this compound [45,58,59]. Based on previous work from others, we also did not test if orexin-B in the DRN alters SPA/EE, nor if OX1R or OX2R is involved in DRN orexin-A-induced SPA using specific antagonists. The current study used orexin-A for DRN injections, which shows potent agonistic activity for both OX1 and OX2Rs, based on previous work indicating a more prominent role of orexin-A vs. orexin-B. One study showed higher SPA following intracerebroventricular (ICV) orexin-A as well as the OX2R receptor agonist ADL-OXB ([Ala11,d-Leu15]-orexin-B) in rats [119]. In Maehara et al.’s study, EMPA (an OX2R antagonist), but not an OX1R antagonist (SB 334867), blocked ADL-OXB-induced hyperlocomotion. In addition, both EMPA and a dual OX1R and OX2R receptor antagonist also blocked orexin A-induced hyperlocomotion, while SB 334867 showed no effects, indicating the possible involvement of OX2R in ICV orexin A-induced locomotor activity [119]. Our earlier study also showed the blockade of ventrolateral preoptic area orexin-A induced SPA by an OX2R antagonist [66], supporting a role for OX2R in SPA. However, another study suggested that the excitation effect of orexin-A on dorsal raphe neurons is via synaptic communication through OX1R but not via OX2R [120]. Similarly, intra-DRN injection of orexin-A significantly reduced the emergence time from isoflurane-induced anesthesia, while injection of orexin-B exerted no significant impact on arousal from anesthesia [121]. One more study demonstrated that both orexin-A and orexin-B excite the serotonergic DRN neurons with equal potency, but OX2 receptors exhibited more pronounced functional significance in the orexin-induced activation of serotonergic neurons [122]. Finally, in the current study, while the distance traveled was low in many mice, the obtained values are in line with our earlier publications and may be a function of the sensitivity of our system. However, activation of the orexin system consistently increased SPA in the current as well as our earlier studies, indicating the SPA-enhancing effect of orexin neuron activation [41,46,117]. To the best of our knowledge, there are no serious side effects associated with the drugs chosen at the given dose (Orexin-A, CNO, muscimol or bicuculline). Even though side effects are reported for bicuculline [123] and muscimol [124] at higher doses, in the current study, we used lower doses identical to those in earlier studies that did not observe any unwarranted side effects following bicuculline [125] or muscimol administration [62]. Even though some studies of orexin overexpression have shown increased fragmentation of sleep/wake states [126], studies from our laboratory have not observed this. In fact, orexin increased the consolidation of sleep/wake states in rats [66]. Orexins also excite neurons important in regulating mood, and having too much or too little orexin activity has been linked to depression and anxiety [127]. However, the chosen dose in the current study was based on our earlier studies, which did not affect anxiety behaviors in rodents (time spent in the center vs. periphery of an open field) [59]. The CNO hydrochloride (CNO) is soluble in aqueous solutions and has no off-target effects in animals lacking DREADD receptors [48]. Accordingly, it is the first choice “designer drug”, with it being an inert and inactive clozapine metabolite [49], and was used in the current study. The dose used in the current study (1 mg/kg) is based on earlier studies from our and other laboratories [41,50]. In sum, the doses chosen for the above drugs did not have any unwarranted side effects, and the doses chosen were based on earlier studies.

## 5. Conclusions

The current study demonstrates that the DRN is an essential brain region that receives orexin inputs with implications for the promotion of physical activity and energy expenditure. Our and other earlier studies have shown higher SPA after orexin-A injections into the paraventricular nucleus, tuberomammillary nucleus, nucleus accumbens, substantia nigra, locus coeruleus, medial preoptic and ventrolateral preoptic hypothalamus [1,66,128,129]. Thus, it is possible that the DRN acts in concert with these areas to enhance SPA and EE following the activation of orexin neurons. The DRN serotonin neurons are the principal neurons that receive orexin afferents from the LH, and the orexin neurons stimulate the DRN. The functional and neuroanatomical findings indicate that the DRN integrates signals associated with bodily energy status to promote appropriate behavioral and physiological responses. Our present results related to the DRN, together with previous reports on orexin’s role in EE and the interaction between the DRN and orexin neurons, reveal that targeting the orexin–DRN axis has the potential to attenuate disorders of energy balance and may provide a target for obesity and diabetes therapy. In addition, by performing these studies in middle-aged animals (13 mo.), where orexin neuron quantity is reduced [46], we believe that modulation of DRN OXA is a potential strategy to mitigate age-induced reductions in SPA and EE.

## Data Availability

Based on institutional guidelines, data will be made available to interested parties upon written request to the corresponding author.
